# Global Temporal and Geographic Stability of Brines on Present-day Mars

**DOI:** 10.3847/psj/abbc14

**Published:** 2020-11-12

**Authors:** Vincent F. Chevrier, Edgard G. Rivera-Valentín, Alejandro Soto, Travis S. Altheide

**Affiliations:** 1 Arkansas Center for Space and Planetary Sciences, University of Arkansas, Fayetteville, AR 72701, USA; 2 Lunar and Planetary Institute, Universities Space Research Association, Houston, TX 77058, USA; 3 Southwest Research Institute, Boulder, CO 80302, USA; 4 Department of Medical Laboratory Science, Eastern Kentucky University, 219 Dizney Building, 521 Lancaster Avenue, Richmond, KY 40475, USA

## Abstract

We combine experimentally verified constraints on brine thermodynamics along with a global circulation model to develop a new extensive framework of brine stability on the surface and subsurface of Mars. Our work considers all major phase changes (i.e., evaporation, freezing, and boiling) and is consistent, regardless of brine composition, so it is applicable to any brine relevant to Mars. We find that equatorial regions typically have temperatures too high for stable brines, while high latitudes are susceptible to permanent freezing. In the subsurface, this trend is reversed, and equatorial regions are more favorable to brine stability, but only for the lowest water activities (and lowest eutectic temperatures). At locations where brines may be stable, we find that their lifetimes can be characterized by two regimes. Above a water activity of ~0.6, brine duration is dominated by evaporation, lasting at most a few minutes per sol. Below a water activity of 0.6, brine duration is bound by freezing or boiling; such brines are potentially stable for up to several consecutive hours per sol. Our work suggests that brines should not be expected near or on the Martian surface, except for low eutectic water activity salts such as calcium or magnesium perchlorate or chlorate, and their (meta)stability on the surface would require contact with atmospheric water vapor or local ice deposits.

## 1. Introduction

Early work considering the formation and stability of liquid water on present-day Mars against atmospheric pressure and temperature suggested that brines would be the most probable stable aqueous phase, given their lower saturation vapor pressure and freezing temperature (Ingersoll 1970). Later efforts using a validated general circulation model (GCM) suggested pure liquid water could be sparingly stable over some 30% of the Martian surface; however, this work also primarily considered air temperature and atmospheric pressure (Haberle et al. 2001); liquid stability against a hyperarid atmosphere was considered but not investigated. Applying their experimental constraints on the stability of ferric sulfate solutions, Chevrier & Altheide (2008) studied the potential global stability of brines against freezing, boiling, and evaporation into a dry atmosphere (i.e., assuming no atmospheric water vapor) using maximum surface temperature constraints from a GCM. However, these constraints did not account for diurnal or seasonal variations of surface conditions (e.g., temperature, humidity, air pressure). Indeed, in situ environmental measurements by the Phoenix lander (Zent et al. 2010; Fischer et al. 2019) and the Mars Science Laboratory (Harri et al. 2014) have now revealed that atmospheric water vapor pressure does not surpass some 2 Pa, and varies significantly throughout the day. To date, work on synthesizing both experimental constraints of brine stability and near-surface Martian environmental conditions into a cohesive global framework of potential aqueous activity has remained limited.

Addressing the presence and stability of liquid brines on the surface of Mars remains a priority for the Mars community (Banfield et al. 2020). Several observations have suggested the possibility of brine-driven or induced geomorphological features on the surface of Mars, such as recurring slope lineae (RSL) or gullies (Malin et al. 2006; McEwen et al. 2011, 2014). In parallel, there is now a significant body of literature on the stability and behavior of brines under Mars-like conditions, which shows that in addition to resisting freezing (Brass 1980), brines are also more resistant to evaporation (Sears & Chittenden 2005; Chevrier & Altheide 2008; Altheide et al. 2009; Chevrier et al. 2009). Moreover, deliquescence has provided a possible pathway for the formation of brines on the surface of Mars (Clark 1978; Gough et al. 2011; Nuding et al. 2014; Gough et al. 2016; Primm et al. 2017), other than melting, considering the global abundance of salts in the shallow regolith. Recently, Rivera-Valentín et al. (2020) used experimental constraints on the deliquescence of Mars-relevant salts along with a GCM to investigate the properties of (meta) stable brines at equilibrium with the ambient atmosphere and found that brines could exist over 40% of the surface for up to 2% of the Martian year.

**Figure 1.**
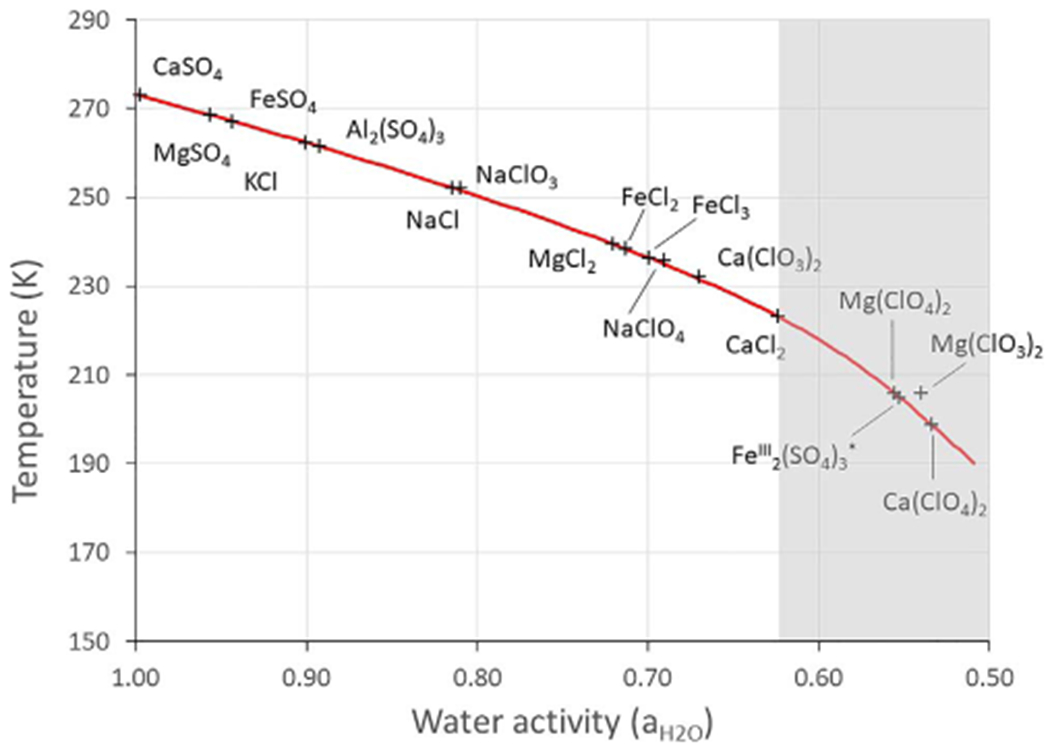
Measured eutectic temperatures as a function of water activity for various salts relevant to Mars (including sulfates, chlorides, perchlorates, and chlorates). The red line corresponds to the theoretical water activity line determined by the ratio of the saturation pressure over ice divided by the saturation pressure over liquid (Murphy & Koop 2005). The gray zone indicates the transition from evaporation dominated stability to freezing and boiling stability on Mars, around *a*_H_2_O_ = 0.62 (Figure 6). Although ferric sulfate has a higher eutectic temperature, around 246.5 K (Hennings et al. 2013), it has been shown that supersaturated brines can easily undergo supercooling and remain liquid down to a glass transition around 205 K (Chevrier & Altheide 2008).

Here, we generalize the work in Rivera-Valentín et al. (2020) to present a new model of brine stability that combines freezing, boiling, and evaporation without restriction on the specific nature of the brine. We do not limit the model to a single value of water activity, but rather investigate a range of water activities from pure water (*a*_H_2_O_ = 1) to the lowest eutectic water activity value relevant to Mars (*a*_H_2_O_ = 0.5, slightly below the water activity of calcium perchlorate, e.g., Figure 1 ). Any known salt eutectic relevant to Mars falls between these two values. Several salts can reach lower water activities, but always at higher temperatures (Nuding et al. 2014). Because of the inverse relationship between temperature and relative humidity (Rivera-Valentín et al. 2018), which at equilibrium is equal to the brine’s water activity, the best brine candidates would have to exhibit low temperatures and low water activities. Therefore, eutectic conditions represent a good compromise between low temperatures and low relative humidity values. The presented results can be applied for any brine at any salt concentration since they rely only on the water activity, independent of the nature of the salt(s) in solution. We use a well-developed and validated global circulation model for Mars: The Mars Weather Research and Forecasting (MarsWRF) model (Skamarock et al. 2005; Richardson et al. 2007) to determine hourly surface temperatures, from which we calculate evaporation rates at the surface and in the subsurface. We also include phase changes, e.g., boiling and freezing, based on the water activity of the brine. Finally, we investigate the effects of atmospheric humidity on the stability and distribution of brines. We mostly focus on the surface of Mars, where brines could be easily detected (directly or indirectly through geomorphological effects), but we also test the effects of layers of regolith on top of brines to place a lower boundary on their stability. This model focuses on brine stability and not formation processes, such as deliquescence or melting and provides a comprehensive framework of liquid stability on present-day Mars.

## 2. Theoretical Background and Modeling

We investigate two scenarios: the surface and subsurface. In both cases, we consider the temporally averaged evaporation rate over a Martian year. On the surface, we consider stability against freezing and boiling. In the subsurface, we consider brine stability at a depth where the temporal temperature variation about the average annual surface temperature is negligible (i.e., some three times the annual thermal skin depth).

### 2.1. Temperature Model

To simulate the subsurface and surface temperatures and the near-surface atmospheric humidity on a global scale, we use the MarsWRF general circulation model. MarsWRF is a generalized and globalized planetary version of the National Center for Atmospheric Research Weather Research and Forecasting (WRF) model (Skamarock et al. 2005; Richardson et al. 2007). MarsWRF has been successfully used to study the Martian atmospheric and climate dynamics (Richardson et al. 2007; Soto et al. 2015; Newman et al. 2017). We used a standard configuration of MarsWRF (see Richardson et al. 2007 for details), with the addition of the recently developed and validated two-moment scheme for microphysical calculations (Lee et al. 2018). This two-moment scheme provides improved simulation of the water cycle, including the radiative effects of water ice clouds, which improves the overall simulation of the Martian climate (Lee et al. 2018). Rivera-Valentín et al. (2020) used MarsWRF and in situ measurements to derive temperatures and water vapor pressures as inputs into deliquescence modeling of brines. We ran MarsWRF for more than five Martian years, using a 5° by 5° horizontal spatial resolution, to ensure that the model was properly spun up and producing a climatology that matches observed Martian climatology. Then, the model was run for one year, outputting the surface temperature, as well as the near-surface atmospheric temperature, humidity, and pressure among other conditions, on an hourly frequency for each grid point in the model. These hourly parameters were then provided to the brine stability model.

### 2.2. Evaporation Model

The evaporation rate of liquid water films into the Martian atmosphere is well described by a diffusion theory (Fick’s equation) modified for the buoyancy of water molecules into the heavier CO_2_ atmosphere (Ingersoll 1970; Sears & Chittenden 2005; Sears & Moore 2005; Chevrier & Altheide 2008); however, this formulation follows three assumptions for simplification. In the derivation, Ingersoll (1970) assumed an isothermal plane above the sublimating or evaporating water (i.e., that the temperature at the surface is equal to the air temperature), a completely dry atmosphere, and did not account for the effects of salts. Here, we do not consider these assumptions, resulting in an evaporation rate given by

(1) $$E=0.17D_{{\text{H}}_{2}\text{O}/{\mathrm{CO}}_{2}}a_{{\text{H}}_{2}\text{O}}\frac{\rho_{\mathrm{sat}}}{\rho_{\mathrm{sol}}}{\left[\frac{(\Delta \rho /\rho )g}{\nu^{2}}\right]}^{\frac{1}{3}},$$

where *D*_H2O/CO2_*D*_H_2_O/CO_2__, is the interdiffusion coefficient of H_2_O_(g)_ and CO_2(g)_; *ρ*_sat_ is the saturation density of water vapor in equilibrium with pure liquid water, which is modified by the brine’s water activity *a*_H_2_O_ to obtain the saturation density above the brine; *ρ*_sol_ is the density of the solution, which leads *E* to represent a rate rather than a flux; Δ_*ρ/ρ*_ is the relative density difference of the gas mixture (water vapor and CO_2_) between the surface of the liquid and the atmosphere; *g* is the gravitational acceleration; and *ν* is the kinematic viscosity of CO_2_. The pure water gas density *ρ*_sat_ is defined as

(2) $$\rho_{\mathrm{sat}}=\frac{p_{\mathrm{sat}M_{{\text{H}}_{2}\text{O}}}}{RT},$$

where *M*_H_2_O_ is the molecular weight of water, *R* is the ideal gas constant and *p*_sat_ is the saturation water vapor pressure above pure liquid water (Murphy & Koop 2005). The buoyancy term Δ*ρ/ρ*, which accounts for the relative density difference between the surface gas above the brine, and the surrounding atmosphere follows

(3) $$\frac{\Delta \rho }{\rho }=\frac{\rho_{\mathrm{atm}}-\rho_{\mathrm{surf}}}{\rho_{\mathrm{surf}}},$$

where

(4) $$\rho_{\mathrm{atm}}=\frac{PM_{\text{C}{\text{O}}_{2}}}{RT_{\mathrm{atm}}}$$

and

(5) $$\rho_{\mathrm{surf}}=\frac{P}{RT_{\mathrm{surf}}}\left(M_{{\text{H}}_{2}\text{O}}\frac{a_{{\text{H}}_{2}\text{O}}p_{\mathrm{sat}}}{P}-M_{\text{C}{\text{O}}_{2}}\frac{\left(P-a_{{\text{H}}_{2}\text{O}}p_{\mathrm{sat}}\right)}{P}\right),$$

where *P* is the atmospheric surface pressure, *T*_surf_ and *T*_atm_ are the temperature at the surface and atmosphere, respectively, and *M*_CO_2__ is the molecular weight of carbon dioxide. Note that assuming *a*_H_2_O_ = 1 and *T*_atm_ = *T*_surf_ reduces Δ*ρ/ρ* to Equation (3) in Ingersoll (1970). In addition, the kinetic parameters are also temperature dependent. The diffusion coefficient of water into gaseous CO_2_ is defined as (Boynton & Brattain 1929)

(6) $$D_{{\text{H}}_{2}\text{O}/{\text{CO}}_{2}}=1.387\times 10^{-5}{\left(\frac{T}{273.15}\right)}^{\frac{3}{2}}\left(\frac{1}{P}\right),$$

where here *P* is in bar. The kinetic viscosity *ν* is empirically defined as (Laesecke & Muzny 2017):

(7) $$\nu =1.48\times 10^{-5}\frac{RT}{M_{{\mathrm{CO}}_{2}}P}\left(\frac{240+293.15}{240+T}\right){\left(\frac{T}{293.15}\right)}^{\frac{3}{2}}.$$

When layers of regolith are covering brine or ice, then the sublimation/evaporation rate is calculated as diffusion through a porous medium following the semiempirical equation (Chevrier et al. 2008)

(8) $$E=\frac{DM_{{\text{H}}_{2}\text{O}}a_{{\text{H}}_{2}\text{O}}p_{\mathrm{sat}}}{LRT\rho_{\mathrm{sol}}},$$

where *E* is the evaporation rate in the atmosphere; *L* is the thickness of the regolith layer; and *D* is the diffusion coefficient of water vapor in the porous regolith. We used an average value of 5 × 10^−4^ m^2^ s^−1^, following previous experimental results (Chevrier et al. 2007, 2008; Hudson et al. 2007; Savijärvi et al. 2020). Here we study the evaporation rate at the depth where the annual thermal amplitude is well attenuated and thus the seasonal cycle becomes negligible. This occurs when the depth is equal to about 3 times the annual skin depth, which is calculated for every location modeled in the GCM using the base thermal inertia map. Therefore, at this depth we use a constant temperature given by the mean annual temperature.

### 2.3. Boiling Effect on Brine Stability

Boiling occurs when the saturation pressure above the brine exceeds the total atmospheric pressure. As the average surface pressure on Mars is ~6 mbar and the triple point of pure liquid water is 273.16 K at 6.11 mbar, every time the temperature is above 273 K the water saturation pressure for pure liquid water exceeds the total atmospheric pressure, and thus liquid boils off the surface (Chevrier & Altheide 2008). Consequently, boiling is a significant constraint for liquids since it is much more efficient at removing water from the surface than evaporation. Brines can avoid boiling because dissolved salts depress the saturation vapor pressure above the liquid, such that the brine’s saturation vapor pressure is *a*_H_2_O_*p*_sat_, allowing them to avoid boiling at much lower temperatures.

### 2.4. Freezing Model

The other process limiting brine stability on the surface of Mars is freezing, which occurs when the environmental temperature drops below the freezing temperature of the brine. This temperature is controlled by the water activity, and therefore, two brines with the same water activity freeze at the same temperature, regardless of their respective composition (Figure 1), as long as they are in equilibrium with water ice (which is the case at the eutectic). For this reason, we use water activity as the most fundamental parameter of the brine. We model the freezing temperature of the brine as a function of water activity by finding the temperature such that *a*_H_2_O_ − (*p*_sat,ice_/*p*_sat,liquid_) = 0. In Figure 1, we compare this approach with the known values for Mars-relevant salts and find that it well approximates their eutectic temperatures.

## 3. Liquid Brine Stability on the Surface of Mars

Before describing the results of the model, we want to highlight that brines described in this model are metastable since at best they are affected by evaporation. Therefore, any reference to “stability” is in fact “metastability.” Only Section 6, which studies the effect of atmospheric water vapor, actually deals with stability in the thermodynamic sense.

**Figure 2.**
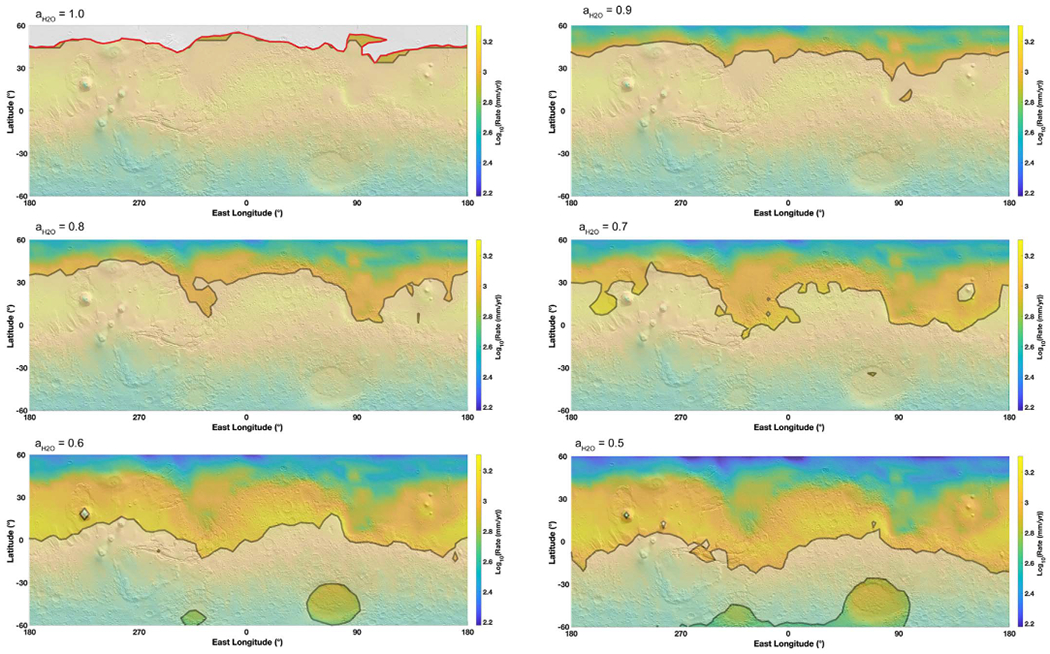
Maps of the evaporation of liquid brines (up to 60° latitude) for various water activities ranging from 1 (pure water) to 0.5 (lowest eutectic brines such as calcium perchlorate), projected on a MOLA shaded relief map. Evaporation rates are calculated in a dry atmosphere (*p*_H_2_O_ = 0) using average surface temperature determined from the global circulation model MarsWRF (Section 2.1) and the theoretical framework presented in Section 2.2. The color maps are then interpolated from the original 5° × 5° grid. Brine stability is also presented with respect to boiling and freezing at the *maximum* yearly temperatures, thus providing an upper limit to brine stability. In the maps, light gray (high northern latitudes at *a*_H_2_O_ = 1) indicates freezing conditions (temperature below the eutectic) while the shaded areas limited by thick black lines (at low latitudes and in the southern hemisphere) indicate boiling conditions (where the water equilibrium vapor pressure *p*_sat_ is above the atmospheric ambient pressure *P*). Liquid brines are only stable in the colored areas, where the maximum temperatures are above the eutectic corresponding to the water activity in the liquid phase and where the saturation pressure does not reach the boiling point.

Figure 2 shows the stability of liquid brines for various water activities representative of different salt solutions. Evaporation rates were calculated hourly and then averaged over an entire Martian year, rather than using the average temperature. Because the evaporation rate is close to an exponential function dependent on temperature, calculating the evaporation rate with the average annual temperature does not result in the average evaporation rate. Moreover, we present annual average values because evaporation is typically considered to be a relatively “slow” process compared with freezing and boiling; however, in some cases, even this approximation is not valid (see Section 5 for more details). Boiling and freezing were also mapped against the maximum yearly temperature, to provide an upper limit to the stability of brines. Therefore, this represents a best-case scenario for brine stability on Mars. In Figure 2, the 5° × 5° resolution of the MarsWRF output has been interpolated and evaporation is represented as a color zone mostly in the mid-to high-latitudes, while boiling appears as a shaded zone in the equatorial regions and southern latitudes (e.g., where temperatures are “high”), and freezing appears as a gray zone, mostly in the high northern latitudes at *a*_H_2_O_ = 1 (e.g., where the temperatures are low).

For high water activities close to *a*_H_2_O_ = 1, (i.e., liquid brines with a low amount of dissolved salts, or poorly soluble ionic compounds such as CaSO_4_ or KClO_4_; see Figure 1), the northern latitudes (above 45°) are permanently frozen (gray zone on Figure 2 at *a*_H_2_O_ = 1). This means that even at the highest temperatures achievable at these locations, stable high water activity liquids could not form. In the equatorial regions, and especially in the southern latitudes, brines can form, but their stability is hindered by boiling (shaded areas in Figure 2). This only leaves a very small region between boiling and freezing where liquid brines can be metastable, although with very high evaporation rates around 10^3^ mm yr^−1^, which is several orders of magnitude above the total condensable water in the atmosphere (Smith 2004).

As water activity decreases (e.g., from 0.9 to 0.7; Figure 2) the stable/evaporation zone in the northern latitudes is significantly extended, down to the equator in some areas (e.g., Isidis Planitia and Chryse Planitia), essentially because of the decrease in the temperature at which boiling occurs. Boiling is affected by altitude and thus surface pressure, and this is the main reason for the general instability of brines around the Tharsis volcanoes and Elyseum Mons (see for example at *a*_H_2_O_ = 0.7). Another significant result is that permanent freezing disappears and brines are potentially stable at the highest annual temperatures. Where liquids are stable against freezing and boiling, the evaporation rates remain around 10^3^ mm yr^−1^, with very little variation with latitude.

Finally, at the lowest possible water activities (0.5–0.6) for salts with the highest solubility (e.g., Ca-chloride, Ca,Mg-perchlorates; see Figure 1), the majority of the northern hemisphere is stable against boiling, while some regions around the major impact basins (e.g., Hellas and Argyre) are also stable in the southern hemisphere, mostly because of the high pressures preventing boiling, as previously observed by Haberle et al. (2001) and Chevrier & Altheide (2008). Liquids at high altitude volcanoes would be unstable due to boiling because of the low total atmospheric pressure. However, evaporation rates are not significantly different from higher activity solutions, except for the highest northern latitudes, which are about 1 order of magnitude lower than at the equator. Equatorial and mid-latitude evaporation rates still remain very high, around 10^3^ mm yr^−1^, but show a sharp decrease by about one order of magnitude close to 50–60°N.

**Figure 3.**
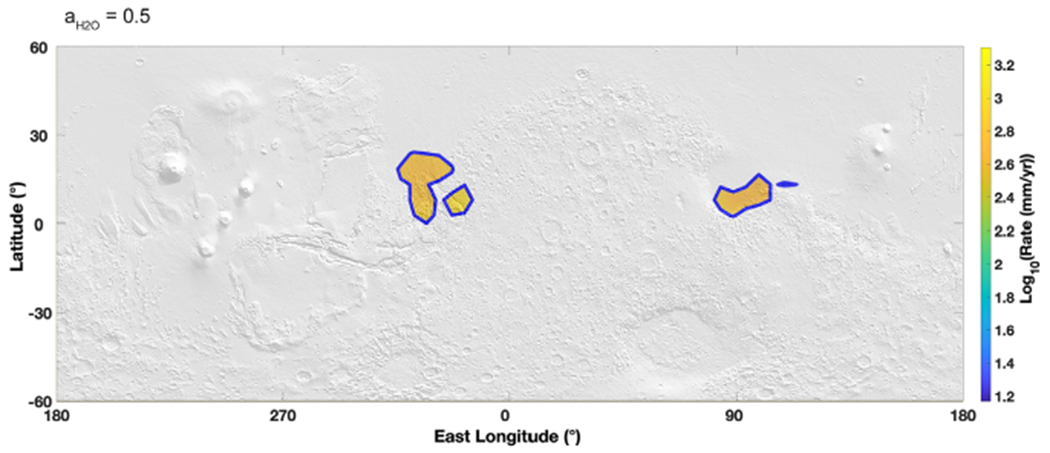
Map of liquid brine evaporation at water activity of 0.5, projected on a MOLA shaded relief map. Evaporation rates are calculated using average surface temperature determined from the global circulation model MarsWRF (Section 2.1) and the theoretical framework presented in Section 2.2. The color maps are then interpolated from the original 5° × 5° grid. Brine stability is presented with respect to freezing at the *minimum* yearly temperature, thus providing a lower boundary to brine stability. Most of the surface is frozen (gray regions) except for the three colored regions delimited by a blue line. Moreover, any brine with a higher water activity is frozen at those temperatures. This shows that only brines with the lowest eutectics in these two regions (south of Chryse Planitia on the west and south of Isidis Basin on the east) can remain permanently liquid at the surface of Mars, with respect to freezing.

By studying brine stability at the lowest temperatures experienced at each modeled location, we also tested for permanent stability (Figure 3). Only brines with the lowest eutectic water activity (~0.5) could be stable against freezing year round, concentrating in three specific regions slightly north of the equator: the southern part of Isidis Planitia on the east, around Aram Chaos and Ares Vallis, and finally the southern part of Chryse Planitia and Simud Vallis on the west. Anywhere else, even a low eutectic brine will freeze at some point during the year. The main reason for the persistence of liquid brines at these minimum temperatures is the high thermal inertia characterizing these regions, which results in “higher” minimum temperatures.

In summary, lowering the water activity results in stabilizing brines by reducing the temperatures at which they will freeze and boil, mostly because both are temperature dependent. Overall, evaporation is not significantly affected, remaining high in equatorial regions and slightly lower at high latitudes. From these observations, ideal regions for brine stability are intermediate to high latitudes, although only brines with low eutectic temperatures can exist at higher latitudes without permanently freezing. Specific regions of interest could include Isidis and Chryse Planitiae, as low eutectic brines are permanently stable given the highest and lowest temperatures at these regions.

## 4. Stability of Liquids in the Subsurface

**Figure 4.**
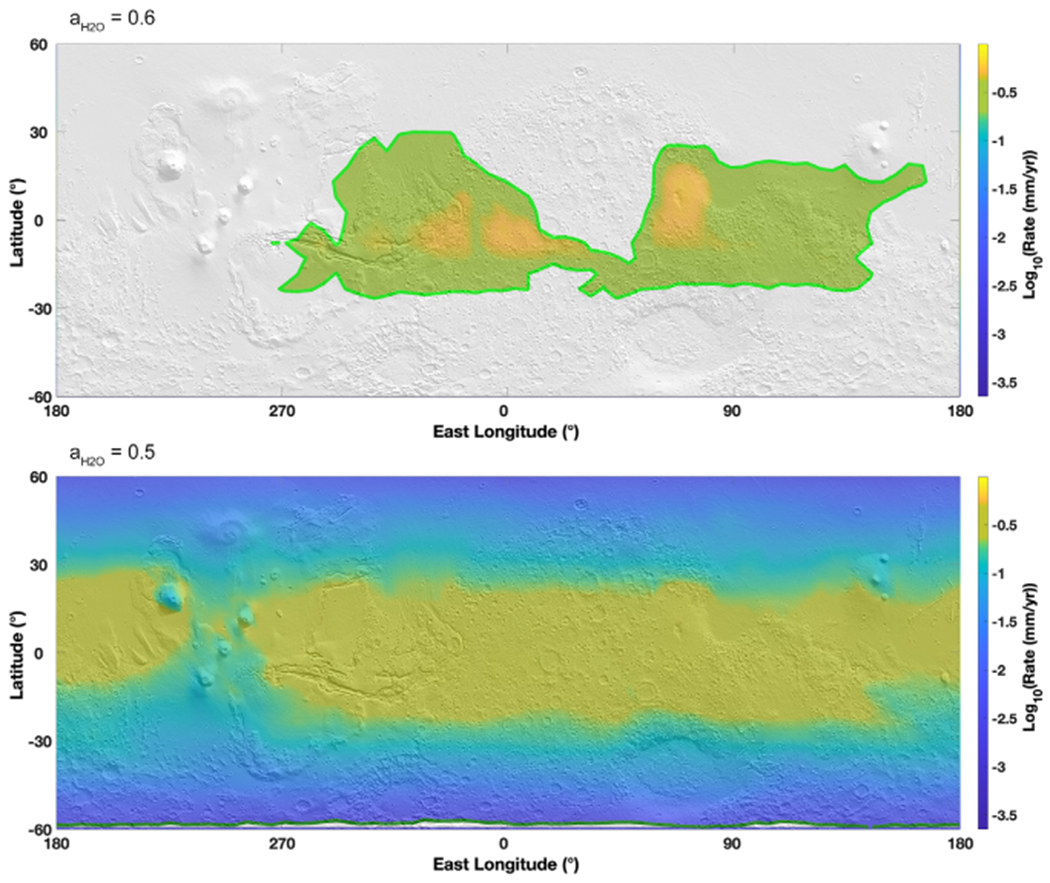
Map of liquid brine evaporation in the subsurface of Mars, projected on a MOLA shaded relief map. Evaporation rates are calculated using average surface temperature determined from the global circulation model MarsWRF (Section 2.1) and the theoretical framework presented in Section 2.2, but modified by diffusion through the regolith at a depth of three times the seasonal thermal skin depth. As the thermal amplitude is completely dampened, we use *average* temperatures to determine freezing. Thus, brines are permanently frozen in the gray areas and liquid only in the colored areas (delimited by a green line). At this depth, there is no boiling because of the overlying regolith. As stability is limited by the average temperature, only brines with water activities of 0.6 or below are stable, while any brine with a higher activity is systematically frozen in the subsurface.

When modeling the stability of brines in the subsurface, the situation is quite different (Figure 4). Under these conditions, the temperature variations are attenuated by the regolith and at around 1–3 m deep the temperature does not fluctuate much from the average annual temperature. Therefore, we used the average annual temperature instead of the maximum annual temperature to define the boundary with respect to freezing. Brine in the gray zone will be permanently frozen and never melt since the temperature does not vary in these locations. On the other hand, brines do not undergo boiling in the equatorial regions because of the overburden pressure and therefore evaporate instead (Figure 4). In addition to evaporation, the second process affecting the stability of liquids is the diffusion-limited water vapor transport through the porous regolith, which significantly reduces the evaporation rates of liquids (Figure 4, Equation (8)). Such diffusion through the regolith results in evaporation rates ~3–4 orders of magnitude lower than at the surface (compare evaporation rates at *a*_H_2_O_ = 0.5 between Figures 2 and 4).

Because average temperatures do not surpass 230 K, this considerably reduces the stability field of liquid brines with respect to water activity. Only salts with the lowest eutectic water activity can be liquid in the subsurface. Salts with a water activity of 0.6 (such as CaCl_2_) are reduced to two small regions centered around the equator (Figure 4, top map). Salts with higher water activities are permanently frozen because there is no variation of temperature across the year. Salts with a water activity of 0.5 (Figure 4, bottom map) are potentially stable over the entire latitude range. There is a sharp transition in the evaporation rates between the high latitudes above 25°–30° and the lower, equatorial latitudes with evaporation rates 1–1.5 orders of magnitude lower, from 10^−0.5^ mm yr^−1^ in the equator to 10^−1^ mm yr^−1^ at 30° latitude and 10^−2^–10^−3^ mm yr^–1^ around 60° latitude. Note that the Tharsis volcanoes, although equatorial, are still characterized by lower evaporation rates because of the lower average temperatures associated with high altitudes.

Globally, subsurface evaporation is ~3–4 orders of magnitude slower than on the surface. Even with such low evaporation rates, evaporation is still high enough that at geological timescales (~10^5^ yr, corresponding to about 10 m evaporated at 30° latitude) current brine activity should not occur in these regions, unless there is a recharge mechanism (Chevrier & Altheide 2008). If some recent process was to form liquid brines, they could be stable for relatively long periods of time (about 10–100 yr for a 1 mm thick film).

## 5. Lifetime of Brines on the Surface of Mars

**Figure 5.**
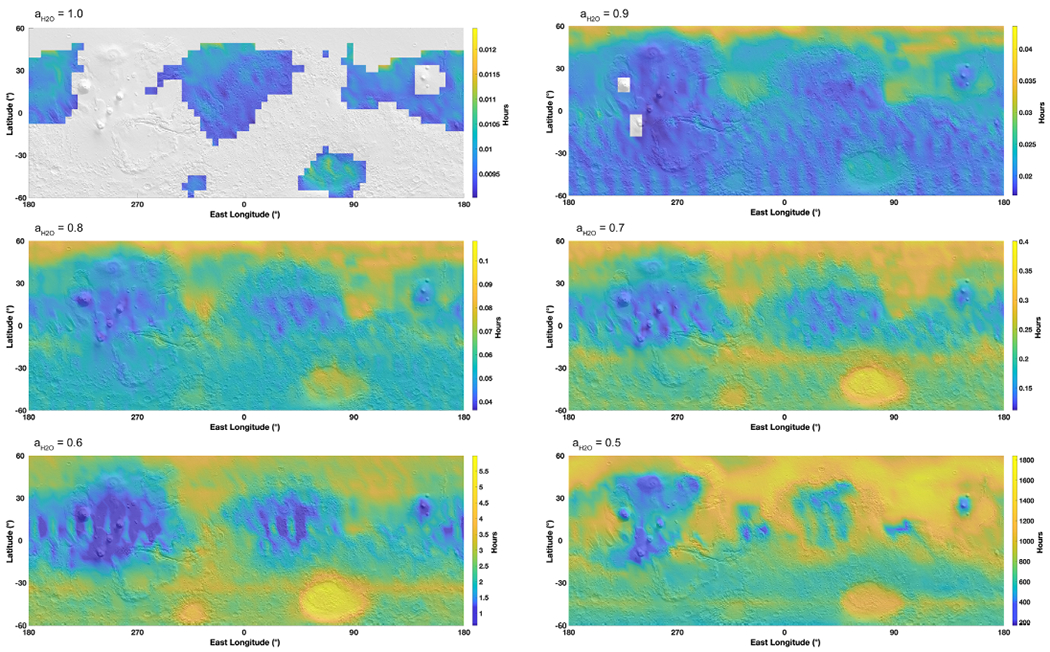
Maps of average brine lifetime on the surface depending on the water activity, projected on a MOLA shaded relief map. The lifetime is based on the average evaporation rates calculated for each hour of the MarsWRF global circulation model output, excluding when freezing and boiling occur. Therefore, gray areas are when liquids are never stable (only freezing or boiling). Note that the maps are not on the same scale due to the wide range of evaporation rates at each water activity (as lower water activities allow for lower temperatures and therefore exponentially lower evaporation rates).

**Figure 6.**
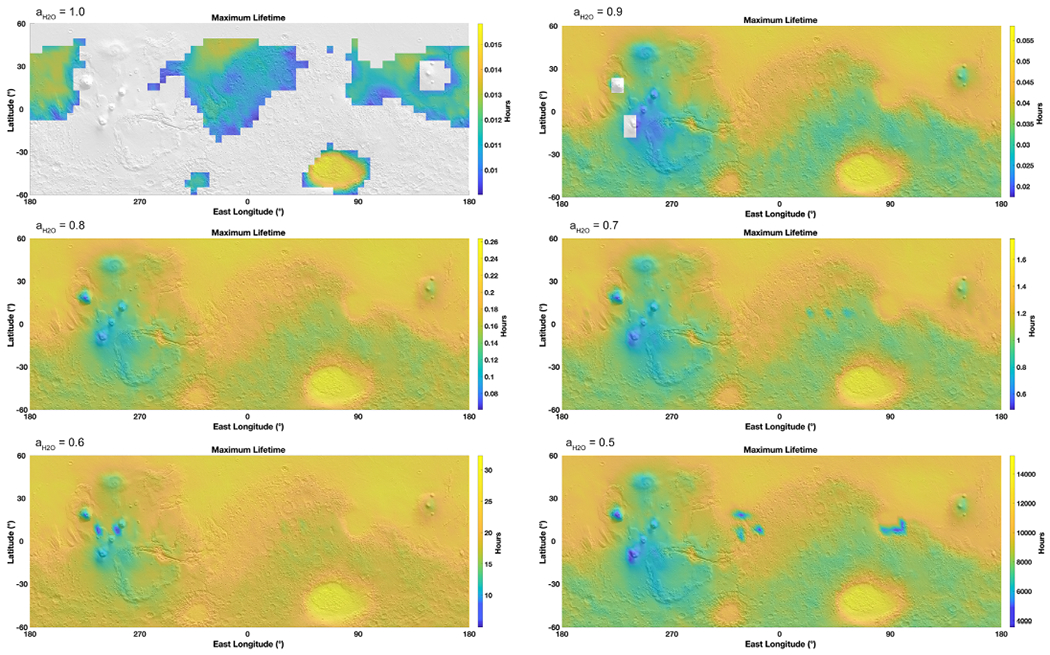
Maps of maximum brine lifetime on the surface depending on the water activity, projected on a MOLA shaded relief map. The lifetime is based on the minimum evaporation rates calculated for each hour of the MarsWRF global circulation model output, excluding when freezing and boiling occur. Gray areas are when liquids are never stable (only freezing or boiling). Note that the maps are not on the same scale due to the wide range of evaporation rates at each water activity (since lower water activities allow for lower temperatures and therefore exponentially lower evaporation rates).

**Figure 7.**
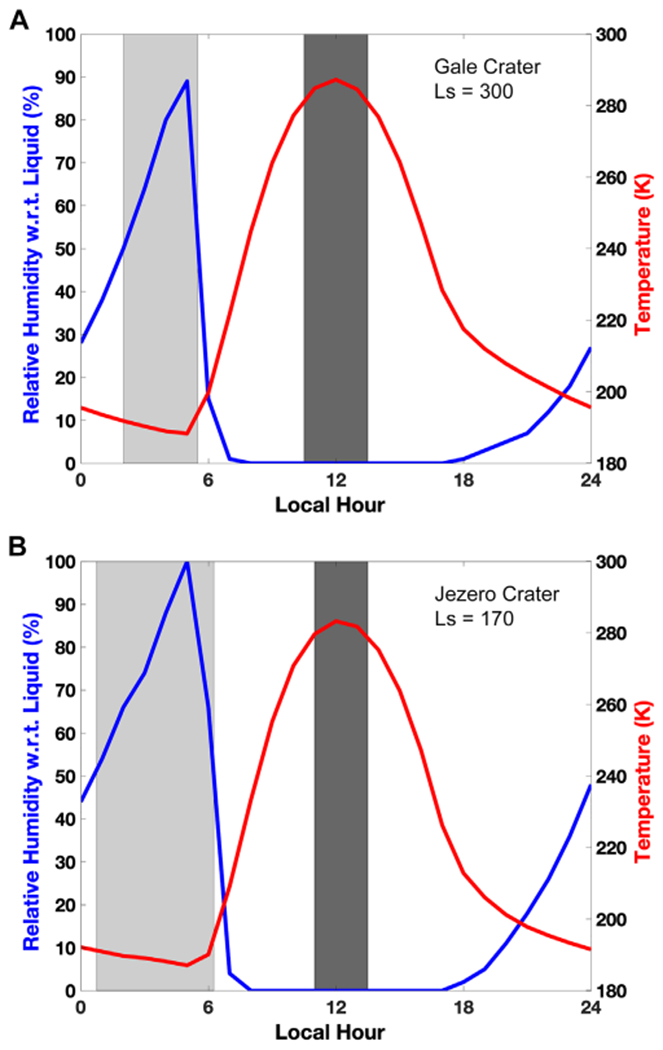
Two examples of diurnal cycle with overlaid brine stability for locations relevant to Mars exploration and a brine activity of 0.5 (eutectic temperature of 190 K, so slightly below calcium perchlorate). A. Gale Crater (Ls = 300) and B. Jezero Crater (Ls = 170). We chose the best-case scenario for brine stability, e.g., resulting in the maximum number of consecutive hours of liquid stability (Ls = 300 for Gale Crater and Ls = 170 for Jezero Crater). In the light gray zone, brines are fully stable with respect to freezing, evaporation and boiling. In the dark gray zone, brines are boiling (see Section 6). There is no freezing because temperatures are high enough to be above the eutectic temperatures.

Following the previous estimates for evaporation framed by boiling and freezing, we evaluate the timescale of brine residence on the surface of Mars. For this calculation, we focused on a 10 *μ*m layer of brine since this thickness is in the same order of magnitude as the water column abundance in the atmosphere (Smith 2004). We determined the evaporation rate over the entire surface of Mars at each hour where the brine would not be frozen nor boiling, over one year. Therefore, these maps are integrated representations of brine stability over an entire year at an hourly resolution, while Figure 2 is a snapshot in time of the best conditions (highest temperature) and Figure 3 at the lowest temperatures. Results show that brines with activities ⩾0.7 only last for a few minutes down to a few seconds (Figure 5). Brines at 0.6–0.5 water activity would last for a few hours at the surface, while those at *a*_H_2_O_ ~ 0.5 could remain present up to about 1000 hr over the course of one year. If using the minimum evaporation rates, giving the maximum lifetime, brines could last up to 10 times longer (Figure 6). Of course, the consecutive environmental conditions required for a brine to be stable for so long do not occur on present-day Mars because of freezing and boiling, which will systematically bind the brine stability over a diurnal cycle (Figure 7). However, these values might be more applicable at the constant temperatures reigning in the subsurface and where boiling does not occur (Figure 4).

**Figure 8.**
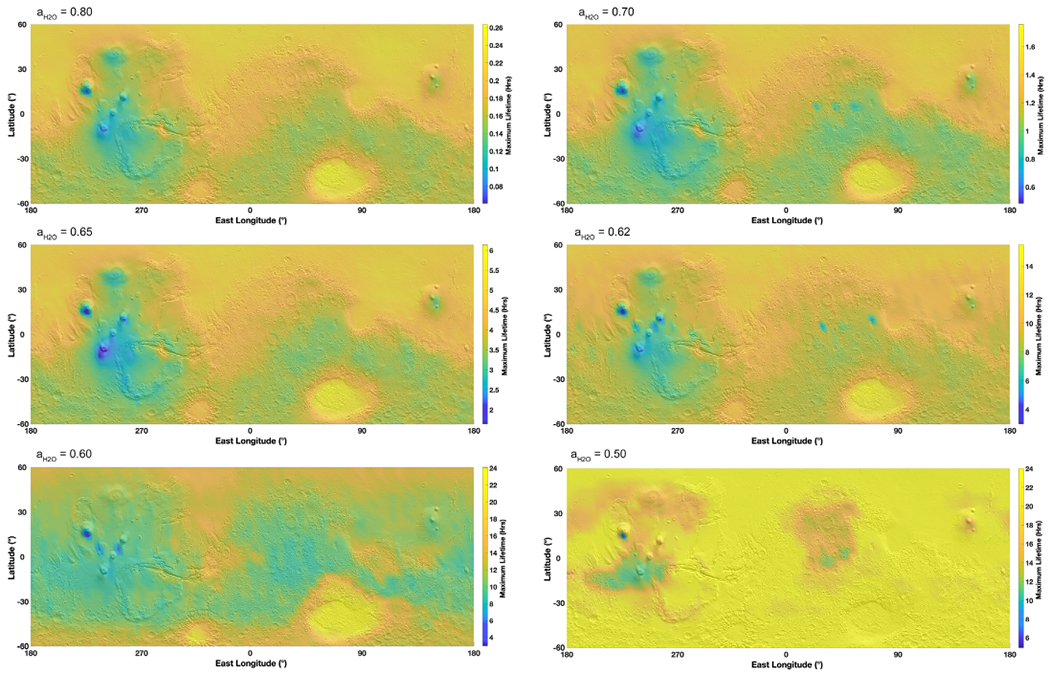
Maps of continuous hours of brine metastability as a function of water activity, projected on a MOLA shaded relief map. These maps are similar to Figure 5, but presenting the duration per sol, so a brine extending to 24 hr is stable over an entire Martian day. Contrary to Figure 5, the minimum evaporation rate (Figure 6) is used to determine an upper boundary for the lifetime of the brine (e.g., best-case scenario). Water activities above 0.8 are ignored as they present a negligible timescale in the order of a few seconds.

**Figure 9.**
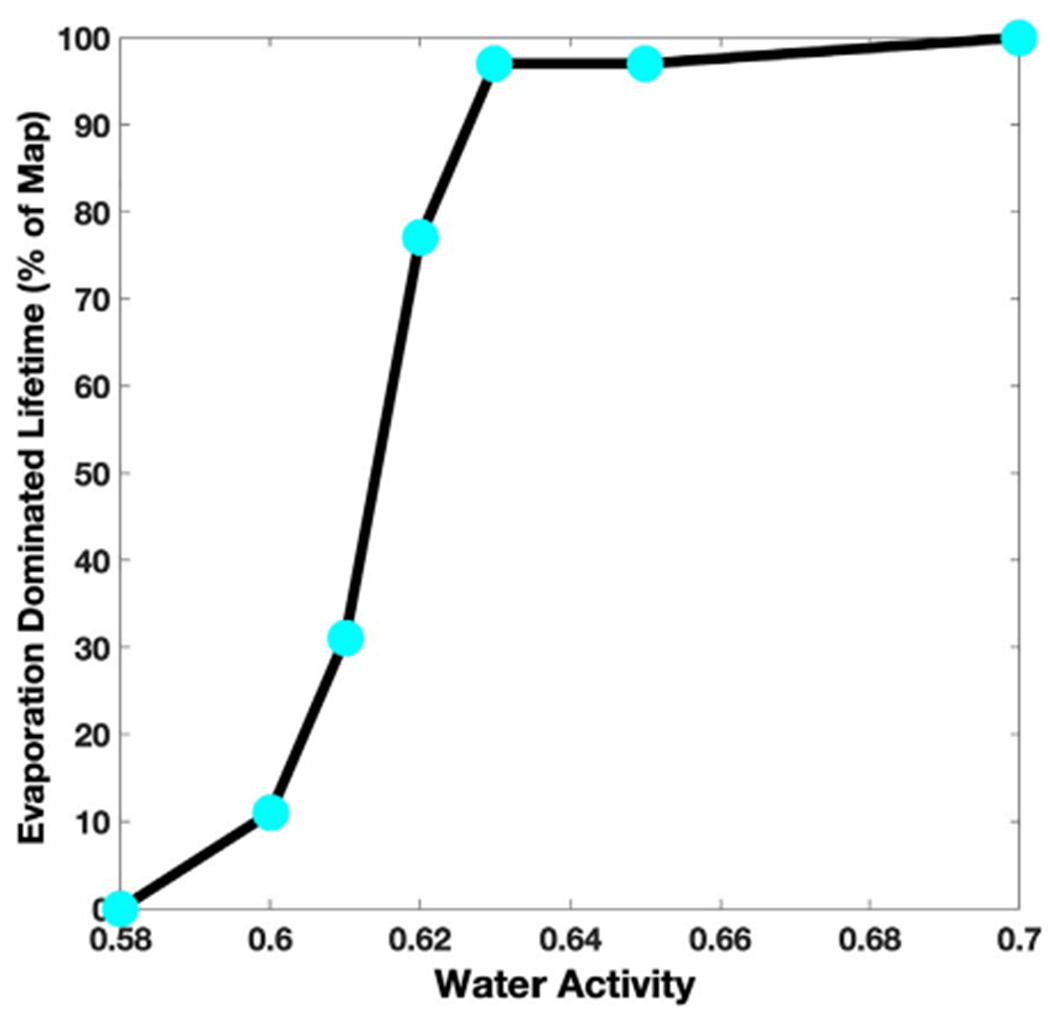
Percentage of the Martian surface where evaporation dominated over boiling or freezing during the lifetime of a brine (based on Figure 5). On this plot, the percent found for a given water activity is shown as a cyan circle and the solid black line is illustrating the trend as a function of water activity. For high water activity brines (i.e., *a*_H_2_O_ > 0.7), evaporation is faster than the hourly changing conditions across all of the surface and thus dominates the lifetime of a brine. Conversely, for *a*_H_2_O_ < 0.58, evaporation is much slower compared to the hourly changing conditions, and so boiling and/or freezing regulate the lifetime of a brine across the surface of Mars, yet are more stable over time.

As a result, we refine the approach by modeling the maximum continuous time before a brine disappears (Figure 8). We frame the stability of brines using freezing and boiling boundaries and determine the maximum time before 10 *μ*m can no longer remain liquid. Brines with water activity above 0.7 will at most last seconds to minutes per sol, while brines with the lowest activities (such as 0.5 or 0.6) can last longer (Figures 7 and 8), up to 24 continuous hours. The most stable regions are in the large impact basins such as Hellas, because of higher pressures limiting boiling at higher temperatures, while brines are never stable at the higher altitudes of the giant volcanoes. These results also show that the stability of higher water activity brines is limited by evaporation, which occurs much faster than the change in environmental conditions that lead to freezing or boiling, while lower water activity brines are limited by freezing and boiling because evaporation has slowed below the diurnal timescale (Figure 9), with a very sharp transition around *a*_H_2_O_ = 0.62.

## 6. Effect of Atmospheric Humidity

**Figure 10.**
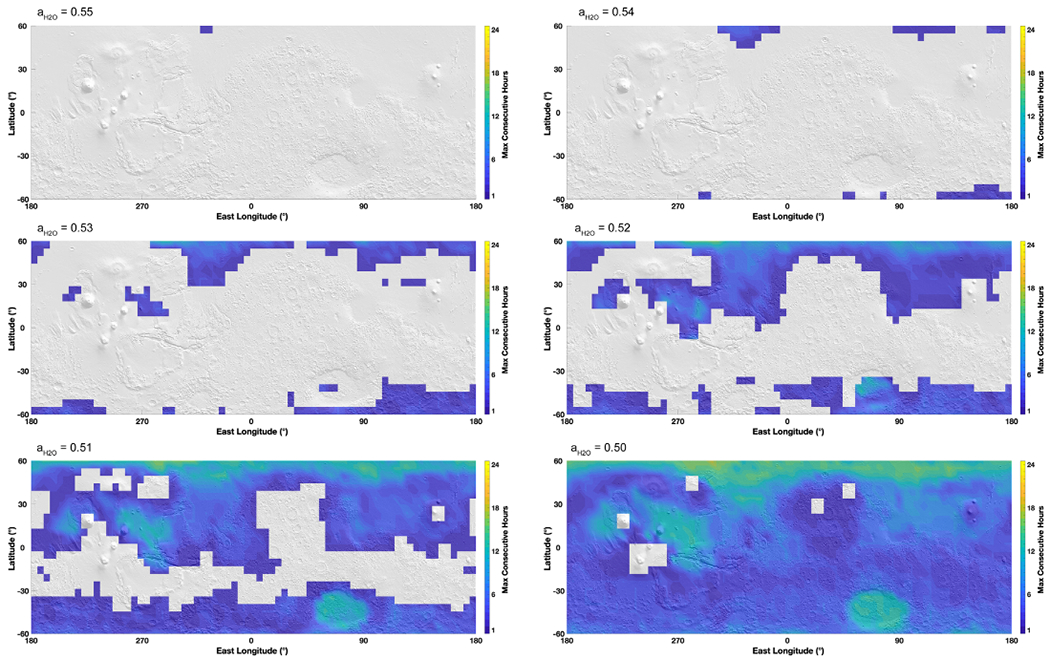
Maps of thermodynamically stable brine duration as a function of water activity, projected on a MOLA shaded relief map. These maps are similar to Figure 5, but include partial pressure of water in the atmosphere. When the water pressure is above the saturation partial pressure of the brine, then the liquid phase is fully stable (e.g., evaporation does not occur). Only brines with water activities below 0.55 are stable on the surface against freezing, boiling and evaporation, while no brine is ever fully stable over an entire day. In specific regions, brines with very low eutectic points can be stable at most for about 12 hr.

An important parameter in the stability and potential formation of brines on Mars is the atmospheric humidity, which can act to slow down evaporation or even completely prevent it if the partial pressure of water vapor is superior to the water saturation pressure above the brine. Deliquescence is a special case, where the starting point is a salt and the atmospheric water vapor is above the saturation vapor pressure for the brine in equilibrium with that salt at the corresponding temperature. To model the lifetime of brines with respect to atmospheric humidity, we determined the number of continuous hours where the brine was not evaporating, boiling or freezing (Figure 7). Only brines with the lowest water activity are stable on the Martian surface, e.g., with *a*_H_2_O_ < 0.55 (Figure 10). No brine is ever stable for a full day, at most for 12–15 hr in Hellas Basin and the northern latitudes above 45°–50°. Interestingly, this is also the typical range allowing for water activities expected for deliquescence on the surface of Mars (Rivera-Valentín et al. 2020).

## 7. Discussion

All the previous calculations show that although nearly all salts could form some liquid solutions, these brines would be unstable to some degree on the surface. Regions with higher temperatures are too dry and evaporation rates are too high so any potential liquid does not last long if formed. Therefore, liquid brines could never be stable for long enough to accumulate and have any significant effect, especially if we consider boiling, which efficiently removes water from the surface. This likely prevents the stable existence of brines in the equatorial regions, except for two interesting regions, Isidis Planitia and Chryse Planitia, where highly concentrated brines (*a*_H_2_O_ close to 0.5) have the potential to remain present all year long because they neither boil nor freeze, even at the lowest temperature. High latitudes (above 45°) and Hellas Basin represent the best regions for liquids to form and eventually be stable, but their lower temperatures also require very soluble salts with associated low eutectic temperatures. Therefore, only a handful of salts have the right properties to form liquids under current Martian conditions (Figure 1). Calcium chloride (CaCl_2_), magnesium and calcium perchlorates (Mg(ClO_4_)_2_ and Ca(ClO_4_)_2_, respectively), magnesium chlorate (Mg(ClO_3_)_2_), and metastable ferric sulfate (Fe^III^_2_(SO_4_)_3_) are the best candidates. All of these salts have been predicted or identified on the surface of Mars by various in situ or orbital methods (Lane et al. 2004; Osterloo et al. 2008; Hanley et al. 2012; Kounaves et al. 2014).

Most of the time, salt deposits are mixtures of several salts. In this case, depending on the composition, the eutectic temperatures will be affected. Salt mixtures have systematically lower eutectic temperature than individual pure phases. This is also true for the deliquescence relative humidity of salts (Gough et al. 2014). Some calculations using the compositions determined by the Phoenix lander Wet Chemistry Lab show that the freezing temperature of the liquid could drop down to 150 K (Elsenousy et al. 2015). Such temperatures are low enough to allow liquid even in the highest latitudes and resist boiling almost anywhere, which would allow for continuous stability over large portions of the surface and extended periods of time (Figures 5 and 10). The problem is that the corresponding amounts of liquid are usually extremely small, because the eutectic composition corresponds to a fraction of the initial salt mixture (assuming the salt mixtures are almost never at the precise eutectic composition). The majority of the initial salts are not soluble enough to contribute to the eutectic mixture. For example, the amount of perchlorate in the regolith at the Phoenix landing site is about 1%, of which likely only a fraction is in the form of Mg, Ca-perchlorate. Thus, the amount of liquid for a 50 wt% solution would remain extremely small, less than 1% of the volume of regolith. Overall, the lower the eutectic the smaller the amount of brine that is formed.

Despite their limited volume in the regolith, due to low eutectics/high concentrations, liquid brines may have important implications for the formation of flow features, such as RSLs or gullies. Both features were assumed to be associated with liquid-triggered or dominated flows (Knauth & Burt 2002; Malin et al. 2006; McEwen et al. 2011; Ojha et al. 2015), before being reinterpreted as dry flows (Dundas et al. 2017; Schmidt et al. 2017). However, these flow features need an activation mechanism related to some form of phase change, and could include deliquescence (Gough et al. 2011; Nuding et al. 2014), melting of ice and salt mixtures (Chevrier & Altheide 2008; Chevrier & Rivera-Valentín 2012), hydration changes (Gough et al. 2020), or even boiling (Masse et al. 2016). Our results show that equatorial regions are too dry (evaporation plus boiling) and high latitudes too cold (freezing). Mid-high latitudes (45°–60°) remain the most active regions in terms of brine phase changes. Depending on brine activity and eutectic temperature, liquid solutions could cycle between melting and boiling, allowing grain movement in the regolith and thus forming RSLs.

**Figure 11.**
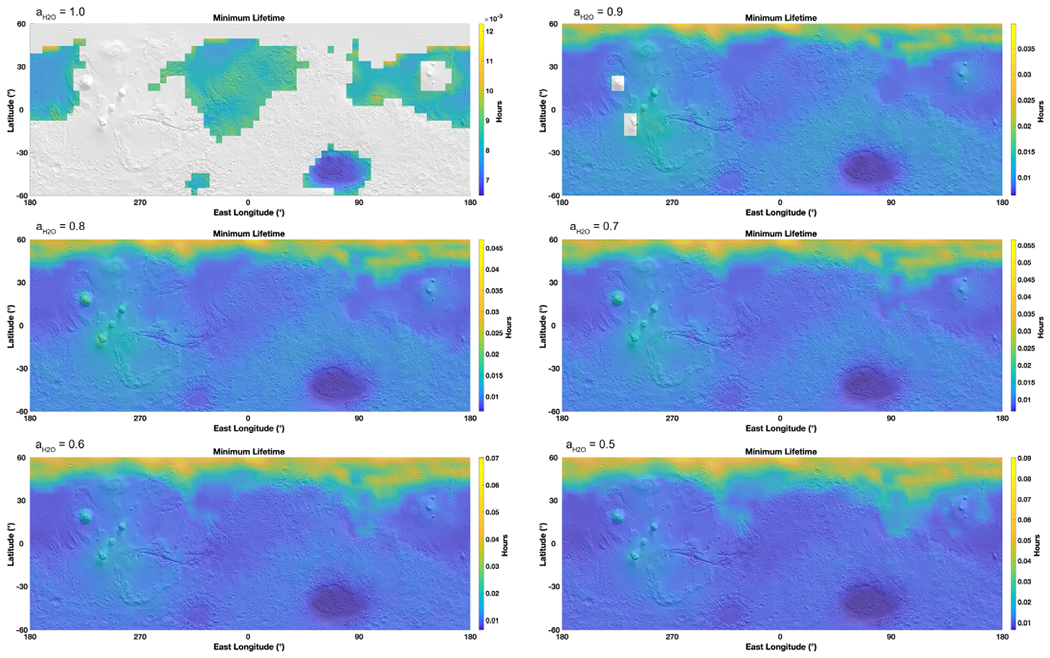
Maps of minimum brine lifetime on the surface depending on the water activity, projected on a MOLA shaded relief map. The lifetime is based on the maximum evaporation rates calculated for each hour of the MarsWRF global circulation model output, excluding when freezing and boiling occur. Gray areas are when liquids are never stable (only freezing or boiling). Note that the maps are not on the same scale due to the wide range of evaporation rates at each water activity (since lower water activities allow for lower temperatures and therefore exponentially lower evaporation rates).

Continuous seasonal formation of RSLs (or any other brine-related process) would require some recharge mechanism in the regolith. Both boiling and evaporation remove water from the surface and transfer it back to the atmosphere. Our maps are determined for the lowest stability by considering a dry atmosphere, but when including humidity the lowest eutectic brines are stable in various regions for up to half a day (Figure 10). During these times, residual brines can absorb atmospheric humidity and recharge. Moreover, when the pressure of water in the atmosphere is above the saturation pressure of the brine, deliquescence occurs. Deliquescence has been extensively studied (Gough et al. 2011; Nuding et al. 2014; Gough et al. 2016) and is likely to occur on Mars (Rivera-Valentín et al. 2018, 2020). However, this mechanism plays a minor role on Mars, as the best estimates show that it would occur for at most 2% of the year and up to a maximum of 6 consecutive hours (Rivera-Valentín et al. 2020). Subsequent fast evaporation at high temperatures (Figure 11) or boiling could quickly remove the brine from the surface, probably at the scale of a few hours (Chevrier et al. 2009), especially since deliquescence is limited by the amount of salt in the regolith (typically around 1% perchlorate or less) and the amount of water vapor in the atmosphere (in the 10 s of precipitable *μ*m). Alternatively, if evaporation is slow enough (Figures 5 and 6), then brines could survive longer enabling them to recharge.

If deliquescence has a limited action, melting could potentially generate more abundant brines, as long as the salt is mixed within the ice matrix or directly in contact with it (Chevrier & Rivera-Valentin 2012). Ice has been observed at mid-latitudes by various direct and indirect methods (Bandfield 2007; Byrne et al. 2009; Dundas et al. 2018). If this ice came in contact with salt deposits, for example during seasonal frost deposition, then melting could be easily induced (Fischer et al. 2016). This mechanism would also be likely to occur in periods of obliquity change, when the ice is redistributed at lower latitudes and temperatures are modified at mid-latitudes (Costard et al. 2002).

Even if brines are limited today in terms of stability, they could still have a significant effect when accumulated over long timescales. Several studies have shown that alteration and diagenesis are possible on present-day Mars, possibly induced by thin films of liquid water (Dickinson & Rosen 2003; Chevrier et al. 2006). The brines formed would be extremely saline (at or close to the eutectic) and highly oxidative/corrosive, being mostly composed of perchlorates, chlorates and chlorides. As a result, these brines could weather the surfaces of rocks and minerals, even as thin films or localized droplets (Rennó et al. 2009).

## 8. Conclusions

We present a global model of brine stability at the surface and shallow subsurface of Mars, covering all relevant chemical compositions and the three fundamental processes affecting their stability: freezing, boiling and evaporation. Because Mars is hyperarid and cold, with very low atmospheric pressure, these processes must be taken into consideration simultaneously to depict an accurate picture of brine stability. These processes tend to have inverse relationships, such as with relative humidity and temperature, which limits the thermodynamic stability of brines, or be on opposite sides of the diurnal cycle, such as boiling at high temperatures and freezing at low temperatures. Therefore, studying these processes separately systematically results in overestimating brine stability on the surface and subsurface of Mars. For example, neglecting boiling will systematically result in equatorial regions appearing stable with respect to liquid.

We find two globally distinct regimes for brine residence on Mars, depending on their overall stability with respect to the processes investigated in this study. Above a water activity of 0.6 (sulfates, most chlorides), brines are highly unstable at the surface and subsurface, limited by fast evaporation and therefore unlikely to ever form in the current climate. Alternatively, below a water activity of 0.6 (some chlorides, chlorates and perchlorates of calcium and magnesium), brines are limited by occasional freezing and boiling, but are more stable on the surface and in the subsurface. In addition to exhibiting far lower evaporation rates (on the order of several days or even weeks, but still bounded by freezing and boiling), we demonstrate that these brines can be consecutively stable for a significant part of the diurnal cycle. The geographical regions most likely to see current brine activity are the high northern latitudes and deep impact basins. In the subsurface, these same brines would be stable in the equatorial regions, where the average temperature is high enough to allow liquid formation. Moreover, there is probably an optimum depth that limits evaporation and suppresses boiling, while allowing significant seasonal melting. These regions represent the most interesting targets for future exploration focusing on present-day habitability and in situ detection of brines.
